# Life-Threatening Airway Obstruction and Septic Shock Due to Submandibular Space Infection: A Case Report

**DOI:** 10.7759/cureus.47181

**Published:** 2023-10-17

**Authors:** Annalee Mora, Alisher Hamidullah, Sophia Samaranayake, Islaam Elnagar

**Affiliations:** 1 Internal Medicine, HCA Healthcare/USF Morsani College of Medicine GME: Oak Hill Hospital, Brooksville, USA; 2 Anesthesiology, HCA Healthcare/USF Morsani College of Medicine GME: Oak Hill Hospital, Brooksville, USA

**Keywords:** nasotracheal intubation, airway obstruction, septic shock, ludwig’s angina, submandibular space infection

## Abstract

Submandibular space infection, a rare and aggressive form of cellulitis, affects the floor of the mouth and neck, potentially leading to life-threatening complications. Although commonly associated with oral trauma and contiguous abscesses, the severity of these odontogenic infections often escalates due to underlying comorbidities. This report presents a unique case of a 74-year-old man who developed severe complications following an outpatient oral procedure. The patient exhibited fever and mouth swelling within a short time, which quickly advanced to impending airway compromise and septic shock. Diagnostic imaging revealed extensive swelling from the left submandibular region extending to the anterior neck and parapharyngeal space, effacing the airway. This necessitated immediate nasotracheal intubation and mechanical ventilation. Medical management comprised emergent antibiotic administration, airway protection, and admittance to the intensive care unit. This case underscores the potential severity of complications arising from an odontogenic infection in the presence of multiple comorbidities following an oral procedure. It emphasizes the need for prompt symptom recognition, emergency airway management, and the initiation of antibiotic therapy. Furthermore, this case illustrates the critical role of various imaging modalities and the choice of intubation technique in patients with an anticipated difficult airway. Despite the severity of submandibular space infection, a timely, effective, and multidisciplinary approach can mitigate fatal outcomes and improve patient prognosis.

## Introduction

In 1836, Ludwig described a rapidly progressing and often lethal infection of the neck’s soft tissues and the floor of the mouth, now known as “Ludwig’s angina” [1]. This infection typically originates from the submandibular space [2] and is most often due to dental infections, tonsillopharyngeal abscesses, physical trauma, sialadenitis, and oral piercings [1]. Clinical signs include edema, tongue displacement, and odynophagia, which may lead to severe complications, such as airway obstruction and septic shock, accounting for a mortality rate of 50% [1,2]. Management strategies include emergent airway protection, antibiotic administration, surgical debridement, adjunctive steroids, and supportive care. We describe a rare case of a life-threatening submandibular space infection in a 74-year-old man with multiple underlying health conditions. He presented with dysphagia, oral edema, fever, and tachycardia less than 24 hours after an oral procedure. The patient showed extensive soft tissue swelling, extending from the left side of his face through the submandibular region and parapharyngeal space to the anterior neck. This case underscores the importance of an interdisciplinary approach in offering emergent interventions, early antibiotics, and supportive care to optimize the outcome in this aggressive and fatal condition.

## Case presentation

A 74-year-old man with a medical history of hypertension, chronic kidney disease stage five, coronary artery disease, and remissive Burkitt’s lymphoma was brought in by emergency medical services. He presented with difficulty swallowing, mouth swelling, fever, and tachycardia after removing his left mandibular tori bone 24 hours earlier at an outpatient dental office. Despite minor mouth swelling, he had no immediate complications from the 15 to 20-minute procedure. However, the next morning, he exhibited bowel incontinence, confusion, agitation, fever, difficulty speaking, and increased oral swelling.

**Figure 1:**
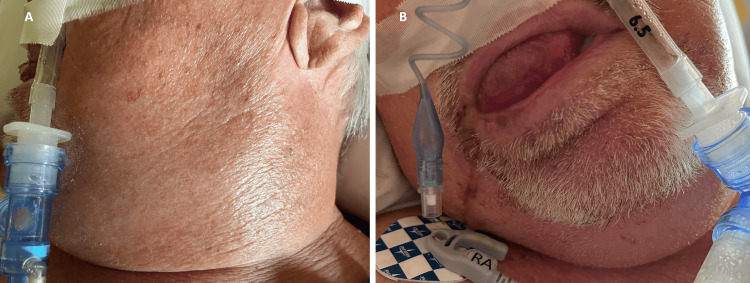
Images of the patient’s oral cavity and neck Panel A shows a view of the patient’s mouth, highlighting the protrusion of the tongue and significant drooling. Panel B presents the patient’s neck, demonstrating significant swelling.

On arrival, his blood pressure was 134/70 mmHg. His heart rate registered 130 beats per minute, and he had a temperature of 39 °C, a respiratory rate of 35, and an oxygen saturation of 96% on room air. Despite being awake and alert, he was in severe respiratory distress. His mouth exhibited drooling and significant swelling (Figure 1A), his tongue was protruding (Figure 1B), and he had difficulty opening his eyes. He communicated solely through nodding. His cardiovascular and respiratory systems examination was unremarkable, and his abdomen was soft and non-tender without organomegaly. His upper and lower extremities showed no edema, with pulses present. An arteriovenous (AV) fistula without a bruit was noted in the right radio-cephalic region.

Due to critical presentation with impending airway compromise, the decision was made to perform an emergent awake fiberoptic nasotracheal intubation, which was successful. The patient was mechanically ventilated, sedated, and immediately started on broad-spectrum antibiotics, fluid resuscitation, and dexamethasone. Nevertheless, the patient’s blood pressure dropped to 85/42 and 58/39 mmHg, necessitating the initiation of norepinephrine and vasopressin to stabilize his hemodynamics. He was promptly admitted to the intensive care unit (ICU) in a state of septic shock.

Laboratory results indicated leukopenia, low hemoglobin, and hematocrit with elevated neutrophils, chloride, blood urea nitrogen, creatinine, and lactic acid (Table 1). His glomerular filtration rate was significantly decreased, and arterial blood gas analysis showed anion gap metabolic acidosis. His electrocardiogram demonstrated sinus tachycardia of 152 without any ST-segment elevations.

**Table 1:** Laboratory investigations

Parameter	Patient Value	Reference Values
White blood cells (x10^3^/µL)	2.9 µL	4.0 – 10.5 µL
Hemoglobin	11 g/dL	13.7 – 15 g/dL
Hematocrit	37%	40 – 51 %
Neutrophils	92.6%	34.0 – 67.9 %
Chloride	114 mmol/L	98 – 107 mmol/L
Lactic acid	4.4 mmol/L	0.4 – 2.0 mmol/L
Serum urea nitrogen	63 mg/dL	7.0 – 18.0 mg/dL
Creatinine	5.3 mg/dL	0.7 – 1.3 mg/dL
Glomerular filtration rate	10.6 ml/min/1.73^m2^	> 60 ml/min/1.73^m2^

**Figure 2:**
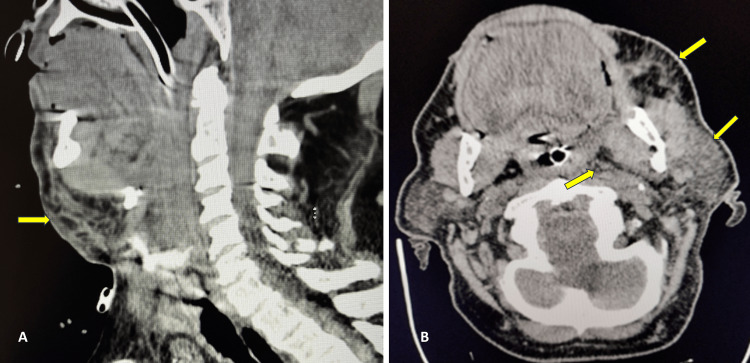
Soft tissue neck CT scans in sagittal (A) and axial (B) views, without contrast, revealing extensive soft tissue swelling extending from the left side of the face and the left submandibular region into the anterior neck There is notable swelling in the parapharyngeal space resulting in effacement of the airway. CT, computed tomography

Chest X-ray revealed no acute cardiopulmonary findings, with an endotracheal tube in place. A facial computed tomography (CT) scan showed submandibular gland adenitis with surrounding cellulitis. A non-contrast neck CT scan showed extensive soft tissue swelling, extending from the left side of his face and the left submandibular region to the anterior neck and into the parapharyngeal space, causing airway effacement (Figures 2A, 2B). These findings raised concerns for Ludwig’s angina. Urgent consultations were made with an oral maxillofacial surgeon and an infectious disease specialist. The surgeon recommended maintaining the patient’s intubation, continuing intravenous antibiotics, and no immediate surgical intervention.

**Figure 3:**
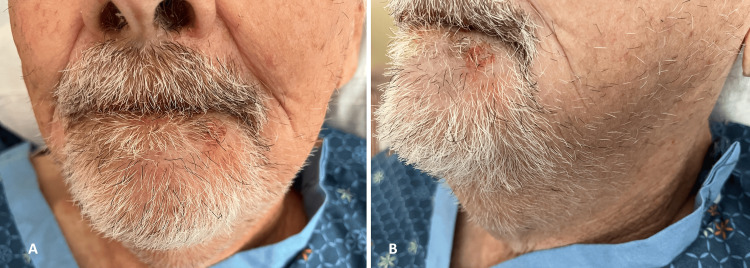
Images illustrating improvements in the patient’s condition Panel A shows a reduction in neck swelling, and panel B displays an improved state of the patient’s oral cavity.

The patient remained intubated in the ICU, with dexamethasone discontinued after 24 hours. His blood pressure began to improve while still on vasopressors. Broad-spectrum antibiotics were continued, and on the second day, his neck swelling decreased although he remained intubated. On the third day, the patient became confused and agitated. He self-extubated and pulled out all his intravenous lines, including the central line, leading to significant blood loss. His hemoglobin levels fell from 9 to 6.8 g/dL, requiring a blood transfusion, and his renal function deteriorated. Nephrology was consulted, and a peritoneal dialysis catheter was inserted because the AV fistula had malfunctioned, and both cephalic veins were calcified and occluded. By the fifth day, his neck and tongue swelling had significantly reduced, allowing him to speak coherently and start eating. After peritoneal dialysis, his renal function improved to baseline levels. By the seventh day, the swelling had greatly diminished (Figure 3). He remained in the hospital for a few more days due to issues with his peritoneal catheter and to complete his intravenous antibiotics course before he was deemed fit for discharge.

## Discussion

Although rare, infections of the submandibular space can be aggressive, rapidly spreading to the floor of the mouth and into the cervical fascia, potentially resulting in life-threatening conditions. Ludwig’s angina, first described by Karl Friedrich Wilhelm von Ludwig in 1836, embodies this threat [1]. This condition is a rapidly spreading, often fatal, cellulitis of the floor of the mouth and neck [3]. Its progression to adjacent areas can lead to airway obstruction, necessitating emergent intervention. Odontogenic infections are the most common cause, accounting for 70% to 90% of cases [4-6]. The premolar is the most frequently implicated site of origin for Ludwig’s angina [7].

Our patient underwent the removal of the left mandibular tori, a bony outgrowth situated on the lingual side of the mandible, typically located in the canine or premolar region [8]. The inflammatory response following this procedure penetrated the sublingual ridge into the submandibular space, leading to significant clinical symptoms in this patient. Other causes of severe submandibular space infection include mouth floor trauma, sialadenitis, mandibular fractures, tonsillopharyngitis, and tongue piercings [3,4,7].

This case report recounts the patient’s rapid development of life-threatening complications, including edema, tongue displacement, odynophagia, airway obstruction, and septic shock within 24 hours following the oral procedure. Due to impending airway compromise, emergency fiberoptic nasotracheal intubation and mechanical ventilation were required. The risk of asphyxiation, the most common cause of death in Ludwig’s angina [6], necessitated this intervention. Patients over 60 or with conditions such as diabetes, obesity, malnutrition, or alcoholism are at a greater risk of critical illness requiring intensive care [1,9]. The patient’s age and underlying comorbidities of hypertension, coronary arterial disease, history of Burkitt’s lymphoma, and stage five chronic kidney disease contributed to the severity of the odontogenic infection. Although the patient has been in remission for six years after undergoing chemotherapy for Burkitt’s lymphoma, the immunosuppressive and myelosuppressive therapies he received pose potential risks for the future development of dental and oral-related complications. These complications may include infection, fibrosis of the neck, jaw, and tongue, salivary gland dysfunction, and disruptions in dental growth and development [10].

Imaging modalities, while not essential for diagnosing submandibular space infection, help assess the extent and location of the inflammation. CT scans are particularly beneficial, and this patient’s scans revealed extensive soft tissue swelling across the left face, left submandibular, and parapharyngeal space, effacing the airway. The sensitivity of a CT scan for Ludwig’s angina is 95%, with a specificity of 53% [11]. A chest X-ray was also performed to exclude potential complications such as mediastinitis.

Following an initial sepsis workup, the patient was started on broad-spectrum antibiotics, ceftriaxone, and vancomycin. This regimen was later switched to piperacillin-tazobactam plus clindamycin to cover a wider range of potential pathogens. Prompt initiation of antibiotic therapy can decrease the mortality rate from 50% to less than 10% [3].

The role of steroids in managing Ludwig’s angina remains controversial. Some studies have indicated the limited utility of steroids [3] while others have suggested that their use may reduce facial and airway edema and enhance antibiotic penetration [11]. Nevertheless, further investigation and more data are necessary to establish definitive conclusions. An oral maxillofacial surgeon was consulted to evaluate the need for surgical drainage. Although a substantial proportion of patients presenting with Ludwig’s angina require incision and drainage, this supplemented procedure is determined based on the presentation of the patient and imaging findings that will guide decisions when necessary. Selected patients can be treated conservatively [12] without the formation of an abscess or gas. Abscess formations in the CT scan will show “rim-enhancing” fluid collections in the sublingual and submandibular spaces. Although the patient’s neck and face CT scan showed extensive swelling in the submandibular and parapharyngeal areas, it did not show a fluid collection suggestive of an abscess. Watchful waiting for up to 48 hours is recommended in such cases, especially if symptoms are identified early, and antibiotics are initiated promptly [5,12]. The patient responded well to immediate management with intravenous broad-spectrum antibiotics to cover anaerobes, gram-positive, and gram-negative bacteria. In our case, the patient did not require surgical intervention and showed improvement in neck swelling within 48 hours.

While in the ICU, the patient developed delirium, a common occurrence in ventilated patients, affecting 60% to 80% [13]. Predisposing factors included the patient’s age, functional impairment, acute illness, medication regimen, and renal failure. Identifying and managing these risk factors is essential to ensure early mobilization, effective communication, spatial orientation, medication review, correction of electrolyte imbalances, and provision of adequate sleep.

## Conclusions

We described a unique case of a 74-year-old man who developed severe complications following an outpatient oral procedure. Prompt recognition and management of submandibular space infections are crucial to prevent rapid deterioration and potentially fatal outcomes. In this case, the immediate actions available to our team in airway protection, infection control, imaging feasibility, and potential surgical intervention were effectively executed. This report highlights the comorbidities contributing to the severity of the patient’s symptoms. While the exact influence of steroids remains unclear, this case suggests a beneficial effect, thereby indicating the need for additional collection and investigation. This condition’s uncommon, life-threatening nature underscores the importance of a collaborative and multidisciplinary approach to achieving optimal outcomes.
